# The predictive value of serum MHR combined with classical metabolic syndrome components in the first trimester for gestational metabolic syndrome: a prospective cohort study in China

**DOI:** 10.3389/fmed.2025.1598363

**Published:** 2025-09-19

**Authors:** Sixu Xin, Linong Ji, Xiaomei Zhang, Yuehan Ma, Xin Zhao, Ning Yuan, Jianbin Sun, Dan Zhao

**Affiliations:** ^1^Department of Endocrinology, Peking University People’s Hospital, Beijing, China; ^2^Department of Endocrinology, Peking University International Hospital, Beijing, China

**Keywords:** metabolic syndrome, monocyte to high-density lipoprotein cholesterol ratio, pregnancy, inflammation, gestational metabolic syndrome

## Abstract

**Objective:**

The objective of the study was to investigate the relationship between inflammatory markers, the serum monocyte-to-high-density lipoprotein cholesterol ratio (MHR) in the first trimester, and gestational metabolic syndrome (GMS), and to identify the risk factors for GMS in early pregnancy and its predictive value.

**Methods:**

This prospective cohort study included 1,410 pregnant women at gestational ages of 7–12 weeks. Pregnant women underwent regular prenatal examinations. Basic information and clinical data of pregnant women were collected. Univariate analysis was performed to identify factors associated with GMS. Variables with a *p*-value of < 0.05 in the univariate analysis were included in the LASSO regression to screen for predictive variables. Multivariate logistic regression was performed to construct the predictive model. A nomogram was constructed based on the predictive variables in the model. The discrimination of the predictive model was evaluated using an ROC curve. Internal validation of the model was performed using the bootstrap method with 1,000 resampling iterations.

**Results:**

Univariate analysis revealed that age, a history of adverse pregnancy outcomes (APOs), body mass index (BMI), systolic blood pressure (SBP), diastolic blood pressure (DBP), fasting blood glucose (FBG), total cholesterol (TC), triglycerides (TG), low-density lipoprotein cholesterol (LDL), white blood cell (WBC) counts, monocyte (MONO) levels, and the MHR in early pregnancy were associated with GMS (*p* < 0.05). Four predictor variables were selected using LASSO regression: MHR, BMI8w, TG8w level, and TC8w. Three multivariable models were developed using GMS as the outcome. Model 1 incorporated predictors selected by LASSO regression as independent variables. Model 2 utilized traditional MS components (BMI8w, TC8w, TG8w, and FBG8w) as independent variables. Model 3 included the MHR, BMI8w, and TG8w as independent variables. The area under the curves (AUCs) were 0.903 (95% CI: 0.862–0.943), 0.896 (95% CI: 0.857–0.935), and 0.895 (95% CI: 0.853–0.938), respectively. The calibrated C-indices for Models 1–3 were 0.898, 0.891, and 0.892, respectively. DeLong’s test results suggested that there were no statistically significant differences in predictive performance among the three models for GMS.

**Conclusion:**

This study has confirmed the predictive value of serum MHR combined with classical MS components in the first trimester for identifying GMS, which could lead to better and earlier identification of GMS patients and provide new ideas for early diagnosis and prevention of GMS.

## 1 Introduction

Metabolic syndrome (MS) is a clinical metabolic disorder characterized by abdominal obesity, insulin resistance, hypertension, hyperlipidemia and abnormal glucose tolerance. Gestational metabolic syndrome (GMS) originates from the gestation period and can lead to adverse pregnancy outcomes (APOs), such as neonatal asphyxia, preterm delivery, and fetal growth restriction. Patients with GMS have a high risk of type 2 diabetes mellitus (T2DM) and cardiovascular disease (CVD) in the long term. GMS can seriously threaten the health of mothers and infants (1). Therefore, it is necessary to analyze and evaluate GMS, a unique aggregation of metabolic abnormalities during pregnancy, to inform further research and academic inquiry. MS is a chronic disease with chronic inflammation at its core, involving inflammatory factors and the body’s immune response. The monocyte-to-high-density lipoprotein cholesterol ratio (MHR) is a novel and simple indicator related to inflammation and oxidative stress status, reflecting the degree of inflammation and oxidative stress in the body. A high MHR has been shown to be associated with increased cardiovascular mortality (2), non-alcoholic fatty liver disease (3), stroke-associated pneumonia (4), and postmenopausal osteoporosis in women (5). However, the relationship between MHR and GMS is still unclear. Therefore, this study aimed to explore the correlation between the MHR in early pregnancy and GMS, evaluate the predictive value of combining the MHR and classical metabolic syndrome components for GMS in early pregnancy, and provide a reference basis for early prevention and diagnosis of GMS and reduction in APOs.

## 2 Materials and methods

### 2.1 Ethics statement

This study was approved by the Ethics Bioethics Committee of Peking University International Hospital. All the protocols followed the ethical guidelines of the institution and national committee and complied with the 1964 Declaration of Helsinki and subsequent amendments. All participants provided written informed consent.

### 2.2 General information

All subjects were enrolled at 7–12 weeks of gestation. The pregnancy and birth outcomes of the enrolled pregnant women and fetuses were regularly followed up. The age, parity, reproductive history, family history, personal history of GDM, hypertension status, and gestational week of the pregnant women were recorded at the time of enrolment. Data on blood pressure, both systolic blood pressure (SBP) and diastolic blood pressure (DBP), as well as measurements of height and weight, were obtained. Body mass index (BMI) was calculated and recorded using the formula BMI (kg/m^2^) = weight (kg)/body height^2^ (m^2^).

The inclusion criteria for the study were as follows: (1) age of 18 years or older, (2) willingness to undergo an oral glucose tolerance test (OGTT) between the 24th and 28th weeks of gestation, (3) planned to receive prenatal checkups and deliver their baby at the hospital, and (4) consented to participate in the relevant questionnaire survey and agreed to the collection of blood samples after being informed about the content of the survey. The exclusion criteria for the study were as follows: (1) MS, diabetes, and hypertension before pregnancy; (2) cardiovascular or cerebrovascular diseases, respiratory diseases, thyroid diseases, blood system diseases, or liver or kidney diseases before pregnancy; (3) pregnant women with infections during pregnancy, multiple pregnancies, and assisted reproductive technology pregnancies; and (4) lack of essential baseline data. Finally, 1,410 subjects with complete data were included in this study.

### 2.3 Biochemical index detection

A total of 5 mL of venous blood was collected from patients in the morning after fasting for 7–12 weeks of their gestational period. The detection indices included white blood cell (WBC) count; platelet (PLT) count; and levels of hemoglobin (HGB), monocytes (MONO), glycosylated hemoglobin (HbA1c), fasting blood glucose (FBG), total cholesterol (TC), triglycerides (TG), high-density lipoprotein cholesterol (HDL-C), low-density lipoprotein cholesterol (LDL-C), alanine aminotransferase (ALT), aspartate aminotransferase (AST), and serum creatinine (SCr). OGTTs were conducted at 24–28 weeks of pregnancy. We measured detection indices for HbA1c, TC, TG, HDL-C, LDL-C, ALT, AST, and SCr levels in the third trimester of pregnancy (the 34th week of pregnancy). We used the subscripts “8w” and “34w” to denote metrics measured in the first and third trimesters, respectively. The biochemical indices were analyzed in the laboratory of Peking University International Hospital Center. HbA1c levels were determined using high-performance liquid chromatography and a Dongcao G8 analyzer. The MHR was calculated using the formula MHR = MONO (10^9^/L)/HDL (mmol/L).

### 2.4 Diagnostic criteria for GMS

Considering the physical characteristics of Chinese individuals, as well as physiological insulin resistance during pregnancy and metabolic changes, the diagnostic criteria for GMS were established based on the 2004 Chinese Diabetes Society (CDS) standards (6) and the findings of Wiznitzer et al. The diagnostic criteria for GMS were as follows: ① Pre-pregnancy overweight (PPOW)/pre-pregnancy obesity (POB), defined as a BMI ≥ 25 kg/m2. ② Elevated blood sugar, diagnosed as gestational diabetes mellitus (GDM): A 75-g OGTT was performed at any time during pregnancy. GDM was diagnosed if any of the following criteria were met: 5.1 mmol/L ≤ FBG < 7.0 mmol/L, an OGTT 1-h blood glucose ≥ 10.0 mmol/L, and 8.5 mmol/L ≤ an OGTT 2-h blood glucose < 11.1 mmol/L. ③ Elevated blood pressure, blood pressure ≥ 140/90 mmHg (1 mmHg = 0.133 kPa). ④ Dyslipidemia, TG ≥ 3.23 mmol/L. Those who possessed at least three of these criteria were diagnosed with GMS. The prevalence of GMS was calculated, and its risk factors were analyzed.

### 2.5 Statistical analysis

SPSS version 31.0 software and R 4.2.1 software were used for data analysis. A t-test was used to compare normally distributed data for quantitative data, while the rank-sum test was used to compare non-normally distributed data between the two groups. Qualitative data are expressed as percentages (%). The chi-square test was used to compare the qualitative data among the groups. Variables with *p* < 0.05 in the univariate analysis were included in the LASSO regression to screen for predictive variables. Multivariate logistic regression was subsequently performed to construct the predictive model. A nomogram was constructed based on the predictive variables in the model. The discrimination of the predictive model was evaluated using a receiver operating characteristic (ROC) curve. Internal validation of the model was performed using the bootstrap method with 1,000 resampling iterations. The differences in the area under the curve (AUC) were compared using DeLong’s test. A *p-*value of < 0.05 was considered statistically significant.

## 3 Results

### 3.1 Comparison of general conditions and biochemical indices between the two groups

Among the 1,410 pregnant women, 214 (15.20%) were diagnosed with GDM. A total of 550 patients (39.00%) were diagnosed with gestational hypertension (GHTN). A total of 145 patients (10.30%) were diagnosed with gestational hyperlipidemia (GHLP). A total of 314 patients (22.30%) were diagnosed with PPOW/POB. Finally, 55 cases met the GMS diagnostic criteria, with women with GDM, GHTN, GHLP, and PPOW/POB accounting for 3.9, 25.70, 10.00, 37.93, and 17.52% of the total study population, respectively. In comparison to the non-GMS group, there was a notable increase in the proportion of individuals with a history of APOs. Compared with those without GMS, women with GMS tended to be older and had higher levels of weight_8w_, BMI_8w_, SBP_8w_ and DBP_8w_ and higher levels of FBG_8w_, TC_8w_, TG_8w_, LDL_8w_, Glu_0min_, Glu_120min_, TG_34w_, and HbA1c_34w_ than those without and also showed higher WBC counts, MONO levels, and MHRs in the first trimester of pregnancy (all *p* < 0.05). Women with GMS also had lower levels of HDL_34w_ and LDL_34w_ and significantly higher PLT_8w_ counts and sCr_34w_ levels than those without GMS (all *p* < 0.05). The levels of HbA1c_8w_, HDL_8w_, HGB_8w_, Glu_60min_, TC_34w_, ALT_34w_, and AST_34w_ did not differ significantly between the two groups. There was no significant difference in the proportion of multipara between the two groups (all *p* > 0.05) (Table 1).

**Table 1:** Comparison of general conditions and biochemical indexes between the two groups.

Variable	Non-GMS group (*n* = 1,355)	GMS group (*n* = 55)	t(X2)	*p*	Variable	Non-GMS group (*n* = 1,355)	GMS group (*n* = 55)	t(X2)	*p*
Age (year)	30.88 ± 3.74	32.80 ± 4.35	−3.71	0.000	WBC(10^9^/L)	8.02 ± 1.89	9.53 ± 2.07	−5.77	0.000
Multipara (%)	55 (3.9)	56 (4.0)	0.02	0.892	MONO(10^9^/L)	0.39 ± 0.11	0.48 ± 0.18	−3.42	0.001
Weight_8w_(kg)	57.39 ± 8.07	67.95 ± 9.03	−9.46	0.000	MHR	0.29 ± 0.10	0.37 ± 0.13	−4.40	0.000
BMI_8w_(kg/m2)	21.71 ± 2.82	26.45 ± 3.04	−12.15	0.000	PLT(10^9^/L)	241.07 ± 53.34	282.24 ± 57.87	−5.59	0.000
SBP_8w_ (mmHg)	109.55 ± 10.31	116.87 ± 12.14	−4.36	0.000	HGB(g/L)	129.72 ± 12.99	133.02 ± 8.52	−1.87	0.062
DBP_8w_ (mmHg)	65.90 ± 9.01	72.33 ± 11.43	−5.01	0.000	GDM (%)	1,196 (84.8)	214 (15.2)	131.86	0.000
HbA1C_8w_(%)	5.20 ± 2.08	5.46 ± 1.09	−0.72	0.470	GHTN (%)	860 (61.0)	550 (39.0)	266.66	0.000
FBG_8w_(mmol/L)	4.90 ± 0.47	5.06 ± 0.56	−2.49	0.013	GHLP (%)	1,265 (89.7)	145 (10.3)	83.41	0.000
TC_8w_(mmol/L)	3.93 ± 0.64	4.41 ± 0.97	−3.67	0.001	GOW/GOB (%)	1,096 (77.7)	314 (22.3)	195.37	0.000
TG_8w_(mmol/L)	0.96 ± 0.51	1.60 ± 0.99	−4.83	0.000	TC_34w_(mmol/L)	6.26 ± 1.14	6.16 ± 1.11	0.62	0.537
HDL_8w_(mmol/L)	1.41 ± 0.26	1.36 ± 0.37	1.08	0.284	HDL_34w_(mmol/L)	1.77 ± 0.33	1.55 ± 0.30	4.84	0.000
LDL_8w_(mmol/L)	2.04 ± 0.52	2.36 ± 0.70	−3.42	0.001	LDL_34w_(mmol/L)	3.38 ± 0.96	3.07 ± 0.95	2.38	0.018
GLU_0min_(mmol/L)	4.90 ± 0.47	5.06 ± 0.56	−6.16	0.000	ALT_34w_(U/L)	12.19 ± 7.20	11.24 ± 5.31	0.97	0.334
GLU_60min_(mmol/L)	7.68 ± 1.63	9.85 ± 1.74	−9.58	0.109	AST_34w_ (U/L)	17.45 ± 5.97	16.09 ± 4.12	1.67	0.095
GLU_120min_(mmol/L)	6.88 ± 1.27	8.35 ± 2.01	−5.39	0.000	Crea_34w_ (umol/L)	47.79 ± 7.72	50.78 ± 9.32	−2.79	0.005
TG_34w_(mmol/L)	3.08 ± 1.15	4.66 ± 2.42	−4.81	0.000	A history of APOs (%)	42 (3.0)	102 (7.2)	7.847	0.005
HbA1C_34w_(%)	5.06 ± 0.84	5.57 ± 0.46	−3.30	0.001					

### 3.2 Analysis of the number of metabolic risk factors clustering during pregnancy and MHR, age, multipara, and a history of APOs

We divided 0, 1 ~ 2, and 3 ~ 4 metabolic risk factors into three groups: N-GMS, pre-GMS, and GMS, respectively. We found sequential increases in serum MHRs and age across the three groups. The differences between the three groups were statistically significant (*p* < 0.05). The proportions of multiparas and those with a history of APOs were greatest in the pre-GMS group, followed by the N-GMS group, and were lowest in the GMS group. The proportions of multiparas were significantly different among the three groups in pairwise comparisons (*p* < 0.05). Compared with the N-GMS and pre-GMS groups, the GMS group had the lowest proportion of patients with a history of APOs, and the difference was statistically significant. Compared with the N-GMS and pre-GMS groups, the GMS group had the lowest proportion of patients with a history of APOs, and the difference was statistically significant (*p* < 0.05) (Table 2).

**Table 2:** Analysis of numbers of metabolic risk factor clustering during pregnancy and MHR, age, multipara, and a history of APOs in the first trimester.

Group	n	Numbers of metabolic risk factor clustering	MHR	Age (year)	Multipara (%)	A history of APOs (%)
N-GMS	636	0	0.27 ± 0.09^bc^	30.42 ± 3.53^bc^	40.70 ^bc^	38.5^c^
Pre-GMS	719	1–2	0.30 ± 0.11^ac^	31.29 ± 3.87^ac^	55.3 ^ac^	54.3^c^
GMS	55	3–4	0.37 ± 0.13^ab^	32.80 ± 3.78^ab^	4.0 ^ab^	7.2 ^ab^

^a^N-GMS group, *P* < 0.05; ^b^ pre-GMS group, *P* > 0.05; ^c^ GMS group, *p* < 0.05.

### 3.3 Variable selection based on LASSO regression

**Figure 1:**
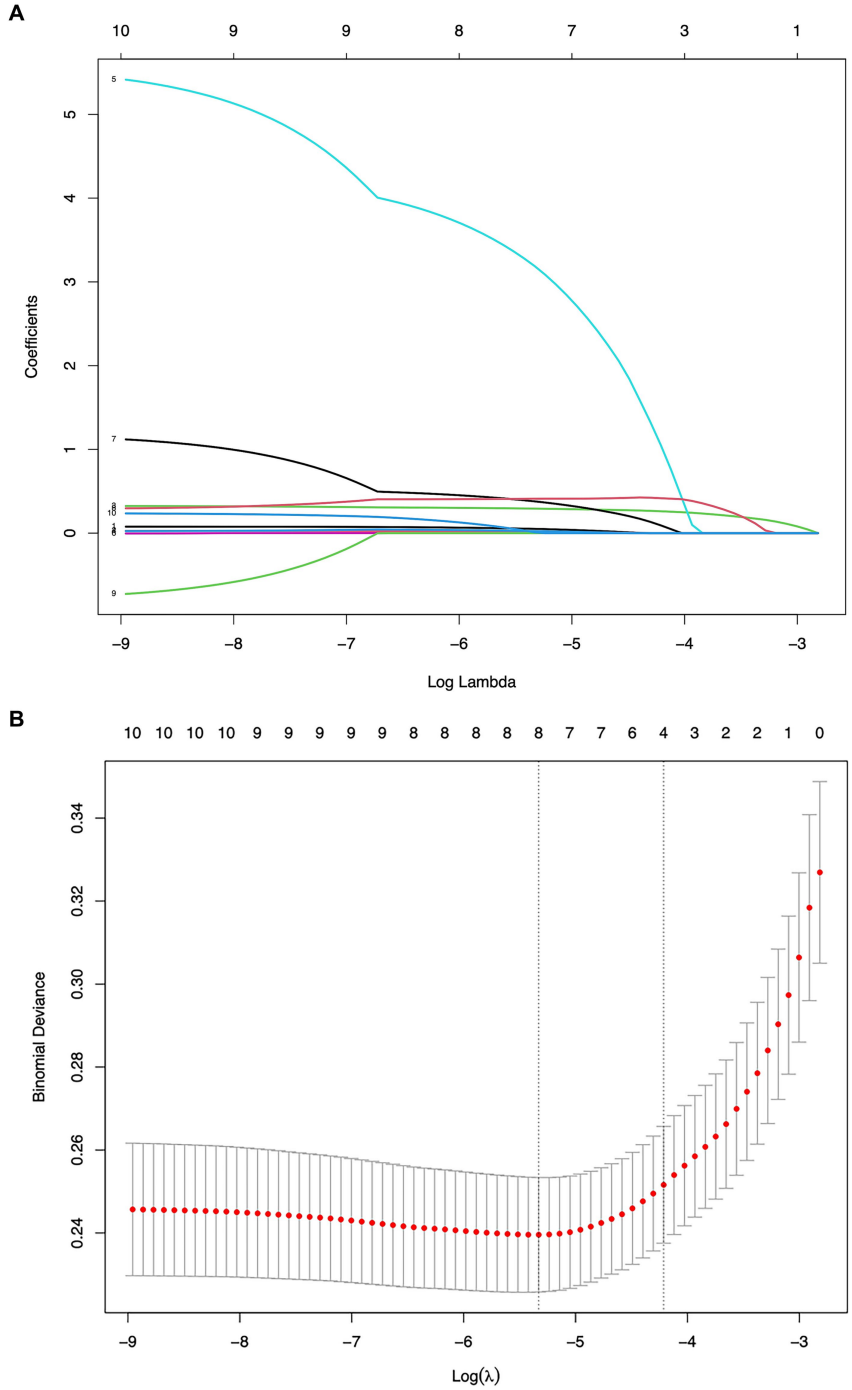
Screening of variables based on LASSO regression. **(A)** The variation characteristics of the coefficient of variables and **(B)** the selection process of the optimum value of the parameter *λ* in the LASSO regression model by the 10-fold cross-validation method.

Variables demonstrating statistically significant differences in early pregnancy through univariate analysis were subjected to LASSO regression with minimum *χ* selection as the optimal criterion, ultimately identifying MHR, BMI_8w_, TG_8w_ level, and TC_8w_ level as predictive factors (Figure 1).

### 3.4 Multivariable logistic regression for predictive model development

**Figure 2:**
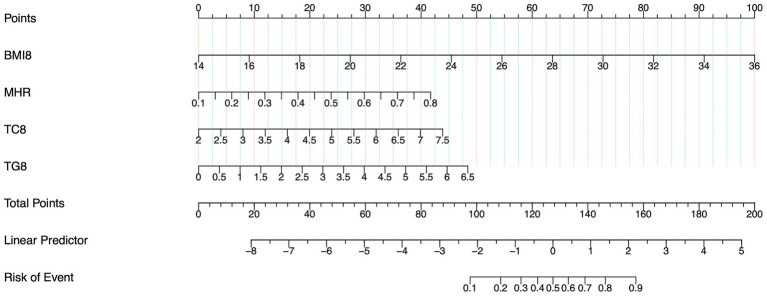
Nomogram of model 1 for predicting GMS in early pregnancy. The sum of the scores on each predictor predicts the probability that GMS will occur.

Three multivariable models were developed using GMS as the outcome. Model 1 incorporated predictors selected by LASSO regression as independent variables. The results demonstrated that the MHR (OR = 80.65, 95% CI: 6.24–1041.50), BMI_8w_ (OR = 1.40, 95% CI: 1.29–1.52), TG_8w_ level (OR = 1.73, 95% CI: 1.20–2.49), and TC_8w_ level (OR = 1.80, 95% CI: 1.21–2.67) were independent predictors of GMS (*p* < 0.05) (Table 3). A nomogram prediction model for GMS was constructed based on these multivariable predictors (Figure 2). Model 2 utilized traditional MS components (BMI_8w_ and levels of TC_8w_, TG_8w_, and FBG_8w_) as independent variables. The analysis revealed that BMI_8w_ (OR = 1.40; 95% CI: 1.29–1.52) and TG_8w_ level (OR = 1.94; 95% CI: 1.38–2.74) were independent predictors of GMS (*p* < 0.05), whereas SBP_8w_ (OR = 1.03; 95% CI: 1.00–1.06) and FBG_8w_ level (OR = 1.00; 95% CI: 0.64–1.55) were not independent risk factors for GMS (*p* > 0.05) (Table 3). Model 3 included the MHR, BMI_8w_, and TG_8w_ levels as independent variables. The MHR (OR = 50.20; 95% CI: 3.97–634.39), BMI_8w_ (OR = 1.40; 95% CI: 1.29–1.52), and TG_8w_ level (OR = 1.97; 95% CI: 1.40–2.78) were significant predictors (*p* < 0.05) (Table 3).

**Table 3:** Predictors for the risk of GMS in the first trimester (*n* = 1,410).

Model	Variables	Estimate	SE	*Z*	*P*	*OR* (95%CI)
Model 1	MHR	4.39	1.305	3.363	0.001	80.65 (6.24–1041.50)
	BMI8w	0.335	0.043	7.835	0.000	1.40 (1.29–1.52)
	TG8w	0.549	0.185	2.919	0.003	1.73 (1.20–2.49)
	TC8w	0.588	0.201	2.96	0.004	1.80 (1.21–2.67)
Model 2	BMI8w	0.338	0.042	7.993	0.000	1.40 (1.29–1.52)
	SBP8w	0.027	0.014	1.93	0.054	1.03 (1.00 ~ 1.06)
	FBG8w	−0.005	0.227	−0.021	0.983	1.00 (0.64–1.55)
	TG8w	0.664	0.176	3.78	0.000	1.94 (1.38–2.74)
Model 3	MHR	3.916	1.294	3.026	0.002	50.20 (3.97–634.39)
	BMI8w	0.338	0.041	8.159	0.000	1.40 (1.29–1.52)
	TG8w	0.68	0.174	3.917	0.000	1.97 (1.40–2.78)

### 3.5 Evaluation and validation of the GMS prediction model

**Figure 3:**
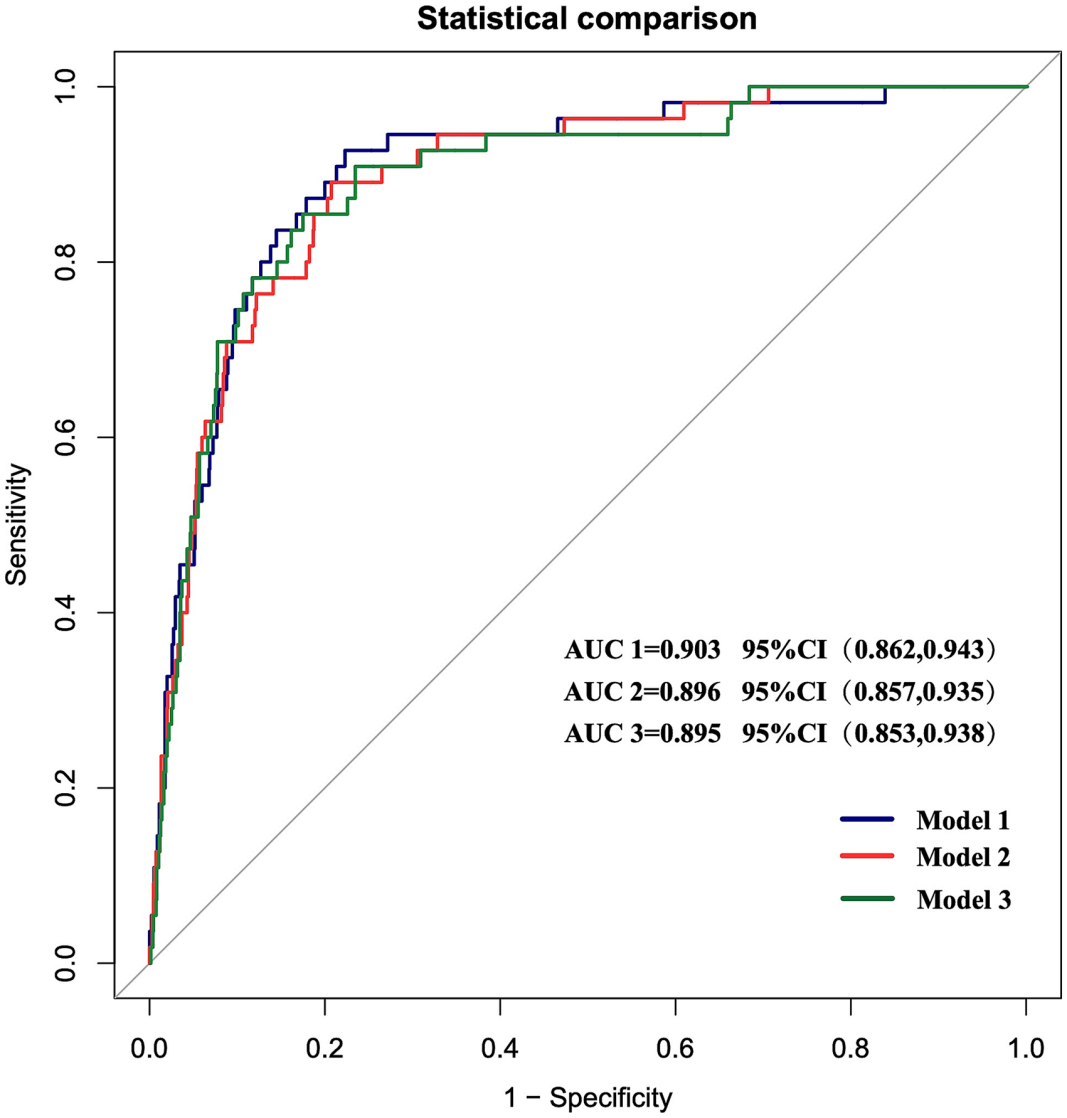
ROC curves of the three prediction models. The AUCs of the three models are 0.903 (95% CI 0.862–0.943), 0.896 (95% CI 0.857–0.935), and 0.895 (95% CI 0.853–0.938), respectively.

The discriminative ability of the three prediction models was assessed by plotting ROC curves. The AUCs were 0.903 (95% CI: 0.862–0.943), with a sensitivity of 92.7% and a specificity of 77.7%, 0.896 (95% CI: 0.857–0.935), with a sensitivity of 89.1% and a specificity of 79.3%, and 0.895 (95% CI: 0.853–0.938), with a sensitivity of 85.5% and a specificity of 82.7%, respectively, for Models 1–3 (Figure 3). Internal validation was performed using the bootstrap resampling method. The calibrated C-indices for Models 1–3 were 0.898, 0.891, and 0.892, respectively, indicating that the models maintained good discriminative and predictive abilities in the bootstrap samples. DeLong’s test results suggested that there were no statistically significant differences in the predictive performance among the three models for GMS (Table 4).

**Table 4:** Validation of model performance and assessment of predictive ability.

Model	AUC (OR 95%CI)	Internal validation via bootstrap method			DeLong’s test
		C-index	_χ_^2^	P*	*p*
Model 1	0.903 (95% CI: 0.862–0.943)	0.898	7.649	0.468	–
Model 2	0.896 (95% CI: 0.857–0.935)	0.891	5.935	0.655	0.456
Model 3	0.895 (95% CI: 0.853–0.938)	0.892	9.814	0.278	0.327

## 4 Discussion

GMS is an independent pathological pregnancy state that includes multiple metabolic abnormalities such as obesity, insulin resistance, elevated blood pressure, and abnormal glucose and lipid metabolism (7). GMS differs not only from traditional single metabolic abnormalities, such as GDM or obesity, during pregnancy but also from simple hypertensive disorder during pregnancy. It can lead to APOs and poses long-term cardiovascular metabolic risks for both mothers and children after childbirth. GMDs are a type of disease that involves two generations and two stages. The occurrence of various metabolic diseases during adolescence or adulthood is related to the intrauterine environment. This phenomenon is called “fetal programming” (8). In recent years, the focus on the origin and prevention of various chronic diseases, including CVD, has increasingly shifted to various adverse environmental exposures in early life. Numerous studies have shown that gestational metabolic disorders (GMDs) not only increase the risk of APOs, such as delayed embryonic development, fetal malformations, spontaneous abortion, preterm birth, macrosomia, large-for-gestational-age infants, dystocia, postpartum hemorrhage, and postpartum infection (9–17), which increase the incidence rate and mortality of pregnant women and perinatal children, but also significantly increase the risk of obesity, diabetes, CVD, and MS among mothers and their offspring in the future, further affecting the long-term health of mothers and children. GDM and GHTN/preeclampsia (PE), as the most common complications during pregnancy, are the main causes of maternal–fetal death and various APOs during the perinatal period. The American Heart Association (AHA) explicitly listed a history of PE and GDM as risk factors for CVD whose risk level was the same as that of traditional CVD risk factors such as obesity, smoking, family history of CVD, and MS (18). Our study revealed that among the 1,410 pregnant women, the prevalence of GDM and GHTN was 15.2 and 39.00%, respectively. Compared with control women, women with GMS tended to be older and had higher levels of weight_8w_, BMI_8w_, SBP_8w_, and DBP_8w_ and higher levels of FBG_8w_, TC_8w_, TG_8w_, and LDL-C_8w_ compared with the control group, which is consistent with previous research findings (1). Compared with those who did not suffer from any GMDs, pregnant women with 1–2 metabolic components had the highest proportion of a history of APOs and multipara, while the GMS group had the lowest proportion. This may be attributed to a higher percentage of primigravidae in the GMS group.

MS is a chronic disease characterized by chronic inflammation involving inflammatory factors and the immune response. As inflammatory cells, monocytes and activated macrophages can produce many adhesion molecules and oxidative mediators. These substances interact with vascular endothelial cells and then absorb and phagocytose oxidized low-density lipoprotein and other lipids through scavenger receptor A and CD-36 to form foam cells, promote inflammation, and accelerate atherosclerosis (19). Our study revealed that compared with control women, women with GMS tended to have higher MONO levels. HDL-C plays important roles in the transport of TG, cholesterol, and phospholipids. HDL-C can inhibit the proliferation of monocytes and the oxidation of LDL-C in vascular walls, leading to anti-inflammatory reactions. Additionally, it can inhibit leukocyte adhesion molecules, pro-inflammatory factors, monocyte activity, and differentiation into macrophages by transporting lipids from peripheral blood and tissues back to the liver. These compounds play important antiatherosclerosis, anti-inflammatory, and antioxidant roles. Our study revealed that compared with control women, women with GMS tended to have lower HDL-C levels. Systemic inflammation and immune status are effective determinants of complete blood count (CBC) and biochemical parameters. These markers are gaining increasing attention in pregnancy monitoring, as they are non-invasive and can be easily calculated. For example, Bozbay et al. demonstrated that the systemic immune-inflammation index (SII, calculated using peripheral platelet, neutrophil, and lymphocyte counts) and the lymphocyte–monocyte ratio (LMR), measured in first-trimester complete blood counts, effectively predict gestational diabetes mellitus (20). The MHR, a novel inflammatory marker that simultaneously reflects both inflammatory injury and protective mechanisms, is attracting increasing attention. Many studies have shown that the MHR, which is a combination of monocyte count and HDL-C level and is closely related to the occurrence and development of atherosclerosis, can be used to predict coronary heart disease and other cardiovascular diseases (21); the MHR can also be used as a prognostic indicator for patients with cardiovascular diseases such as primary hypertension, asymptomatic abdominal aortic aneurysm, isolated coronary artery dilatation, coronary atherosclerosis, and acute coronary syndrome (22, 23). Patients with infective endocarditis have a higher MHR, indicating that the MHR can be used as a predictive indicator of systemic inflammation (24). Our study revealed that women with GMS tended to have higher WBC counts, MONO levels, and a MHR during the first trimester of pregnancy. Furthermore, the MHR increased progressively with the accumulation of metabolic components, a statistically significant difference that correlated with an enhanced inflammatory state in the body.

In this study, a nomogram model for predicting GMS was developed, and LASSO regression was used to identify four predictive variables: the MHR, BMI_8w_, TG_8w_ level, and TC_8w_ level. The model demonstrated an AUC of 0.903, with a sensitivity of 92.7% and a specificity of 77.7%. Internal validation was performed using the bootstrap method with 1,000 resamples, which demonstrated that the model possesses good discriminative ability. Currently, there are no globally unified diagnostic criteria for gestational metabolic syndrome. Furthermore, we constructed a second predictive model using logistic regression analysis based on the four classical metabolic components of MS: BMI_8w_, FBG_8w_, TC_8w_ level, and TG_8w_ level. The results showed that this model achieved an AUC of 0.896, with a sensitivity of 89.1% and a specificity of 79.3%. To reduce predictive error and simplify the model, a third predictive model was developed based on three variables selected via LASSO regression: MHR, BMI_8w_, and TG_8w_ levels. The results indicated that this model achieved an AUC of 0.895, a sensitivity of 85.5%, and a specificity of 82.7%. DeLong’s test results suggested that there were no statistically significant differences in the predictive performance among the three models for GMS. The prediction model constructed on the basis of the four predictor variables selected by LASSO regression had the highest AUC and sensitivity, indicating strong predictive performance for GMS risk and offering valuable guidance for clinicians to make accurate judgments.

## 5 Conclusion

In summary, the serum MHR during the first trimester has diagnostic value for GMS and is an independent risk factor for GMS among pregnant women. This study has confirmed the predictive value of the serum MHR combined with classical MS components in the first trimester for identifying GMS, which could lead to better and earlier identification of GMS patients, provide new ideas for early diagnosis and prevention of GMS, and provide certain clinical references for the pathogenesis of GMS. It is also helpful for screening high-risk populations. However, this study has several limitations: 1. This was a single-center, prospective cohort study with a relatively small sample size, which may introduce potential biases and confounding factors. These limitations could also result in the suboptimal performance of the predictive model when applied to larger populations. 2. This study was limited to data collected from a single center and lacked external validation. To enhance the robustness of the inferences, the bootstrap method was applied with 1,000 resampling iterations. Future efforts should focus on expanding the sample size within our center and incorporating multicenter data for external validation to improve the model’s performance and generalizability. 3. This study did not investigate the specific inflammatory mechanisms underlying GMS, which warrants further investigation in future research.

## Data Availability

The raw data supporting the conclusions of this article will be made available by the authors, without undue reservation.
